# Tau isoforms are differentially expressed across the hippocampus in chronic traumatic encephalopathy and Alzheimer’s disease

**DOI:** 10.1186/s40478-021-01189-4

**Published:** 2021-05-12

**Authors:** Jonathan D. Cherry, Camille D. Esnault, Zachary H. Baucom, Yorghos Tripodis, Bertrand R. Huber, Victor E. Alvarez, Thor D. Stein, Dennis W. Dickson, Ann C. McKee

**Affiliations:** 1 VA Boston Healthcare System, 150 S. Huntington Ave, Boston, MA 02130 USA; 2 Department of Pathology and Laboratory Medicine, Boston University School of Medicine, Boston, MA 20118 USA; 3 Department of Neurology, Boston University School of Medicine, Boston, MA 20118 USA; 4 Boston University Alzheimer’s Disease Research and CTE Center, Boston University School of Medicine, Boston, MA 20118 USA; 5 Department of Biostatistics, Boston University School of Public Health, Boston, MA 20118 USA; 6 VA Bedford Healthcare System, Bedford, MA 01730 USA; 7 National Center for PTSD, VA Boston Healthcare System, 150 S. Huntington Ave, Boston, MA 02130 USA; 8 Department of Neuroscience, Mayo Clinic, Jacksonville, FL 32224 USA

**Keywords:** CTE, AD, Tau isoforms, Hippocampus, 3R, 4R

## Abstract

Chronic traumatic encephalopathy (CTE) is a progressive neurodegenerative disease, characterized by hyperphosphorylated tau, found in individuals with a history of exposure to repetitive head impacts. While the neuropathologic hallmark of CTE is found in the cortex, hippocampal tau has proven to be an important neuropathologic feature to examine the extent of disease severity. However, the hippocampus is also heavily affected in many other tauopathies, such as Alzheimer’s disease (AD). How CTE and AD differentially affect the hippocampus is unclear. Using immunofluorescent analysis, a detailed histologic characterization of 3R and 4R tau isoforms and their differential accumulation in the temporal cortex in CTE and AD was performed. CTE and AD were both observed to contain mixed 3R and 4R tau isoforms, with 4R predominating in mild disease and 3R increasing proportionally as pathological severity increased. CTE demonstrated high levels of tau in hippocampal subfields CA2 and CA3 compared to CA1. There were also low levels of tau in the subiculum compared to CA1 in CTE. In contrast, AD had higher levels of tau in CA1 and subiculum compared to CA2/3. Direct comparison of the tau burden between AD and CTE demonstrated that CTE had higher tau densities in CA4 and CA2/3, while AD had elevated tau in the subiculum. Amyloid beta pathology did not contribute to tau isoform levels. Finally, it was demonstrated that higher levels of 3R tau correlated to more severe extracellular tau (ghost tangles) pathology. These findings suggest that mixed 3R/4R tauopathies begin as 4R predominant then transition to 3R predominant as pathological severity increases and ghost tangles develop. Overall, this work demonstrates that the relative deposition of tau isoforms among hippocampal subfields can aid in differential diagnosis of AD and CTE, and might help improve specificity of biomarkers for in vivo diagnosis.

## Introduction

Tau is a microtubule associated protein that under homeostatic conditions, facilitates axonal transport and supports microtubule stability [28, 42]. Under pathologic conditions, tau becomes unbound from microtubules, hyperphosphorylated at specific sites, and begins to aggregate into pathological oligomers [16]. The deposition and subsequent spread of tau is a characteristic feature to a class of neurodegenerative diseases termed “tauopathies”. The most common tauopathy is Alzheimer’s disease (AD) which arises secondary to amyloid beta toxicity. There are a several primary tauopathies such as chronic traumatic encephalopathy (CTE), primary supranuclear palsy (PSP), corticobasal degeneration (CBD), primary age related tauopathy (PART), Picks disease, and age related tau astrogliopathy (ARTAG). Although tau is a central feature for each disease, there is considerable variation of clinical symptoms, age of disease onset, brain regions affected, and even differences in the tau protein itself. Recent cryo-electron microscopy (cryo-EM) studies have demonstrated different tau fibril structures between AD, CTE, and CBD [13, 14, 43]. Furthermore, tau can be hyperphosphorylated at dozens of sites resulting in unique profiles [41]. Obtaining a better understanding of the intricacies that exist between the different tauopathies is critical because of its potential to reveal unique mechanisms that might serve as therapeutic targets and additionally, provide more resolution for biomarker discovery to identify neurodegenerative disease in life.

The microtubule-associated protein tau (MAPT) gene produces six different isoforms of tau through alternative splicing of pre-mRNA of exon 2, 3, and 10 [3]. Exclusion of exon 10 results in isoforms with three microtubule binding domain repeats (3R) while inclusion results in isoforms with four repeats (4R) [25]. Each isoform is present in the human central nervous system and is believed to have different physiological functions. 3R tau predominates in brain development, but 4R tau increases during early life and reaches equal levels to 3R in adulthood [19, 32]. Tauopathies also demonstrate differential expression in the pathological aggregates of either 3R or 4R tau and help further define some diseases. Disease such as PSP and CBD primarily express 4R tau, while Pick’s disease express 3R tau [4]. AD, CTE and PART have all been found to express both 3R and 4R tau [8, 9, 37]. However, the isoform expression might be dynamic and change over the course of disease. In CTE, early pathology was observed to express more 4R tau while later, more severe pathology had equal or higher levels of 3R tau suggesting an evolution of isoform during the course of disease [8]. Similar evolution of tau has been suggested for AD as well [18, 39]. It was hypothesized that the elevated 3R density was correlated with the appearance of extracellular neurofibrillary tangle (NFT) pathology that was left behind after a neuron has died (ghost tangle) [8, 38].

CTE is a progressive neurodegenerative disease that is found in individuals with a history of exposure to repetitive head impacts. The pathognomonic lesion consists of hyperphosphorylated tau in neurons arranged around blood vessels at the depths of the cortical sulcus. In early stage disease, pathology is typically restricted to the neocortex. However, in more advanced stages, tau is observed to spread to the medial temporal lobe. This is in contrast to AD, where early stage pathology is found in the entorhinal cortex, while the neocortex is affected in later stages. As similar regions are affected in both diseases, this presents a challenge for differentiating between AD and CTE when pathology becomes severe. Additionally, neuritic beta-amyloid plaques can be found in around one third of cases with CTE, resulting in further complexity [36]. Therefore, increased ability to distinguish between neuropathologic features in commonly affected regions are critical.

Although the hippocampus is heavily affected in AD and CTE, several studies have documented variable regional vulnerability among the individual hippocampal cornu ammonis (CA) subfields. AD might preferentially affect the subiculum and CA1, while CTE preferentially affects CA4 and CA2 [15, 29]. However, a detailed comparison directly comparing the hippocampal tau burden between AD and CTE has yet to be accomplished. This analysis can be further refined to compare 3R and 4R tau levels and determine if more complex changes are occurring than previously observed with routine tau immunohistochemistry.

Given the complexity and heterogeneity of tau across multiple disease, there is critical need for more large-scale comparative studies that analyze the tau isoform and regional deposition between similar neurodegenerative diseases. In this study 41 case of CTE and 50 cases of AD were used. Multiplex immunofluorescence analysis was used to measure 3R and 4R tau isoforms across the hippocampal subfields, in addition to the temporal cortex grey and white matter. Analysis of the 4R/3R ratio, primary subfield affected, and direct pairwise comparisons of each subfields between diseases was performed. Finally, the 4R and 3R densities were compared to measures of ghost tangle pathology to determine if there were functional consequences to specific isoform deposition. Overall, this work leveraged expertise in tauopathies to better understand the nature of tau in AD and CTE, and to determine if unique expression patterns exist that might aid in neuropathological assessment, act as novel biomarkers, or provide insight into possible therapeutic targets.

## Methods

### Subjects

Post-mortem formalin fixed human brains tissue was obtained and processed from 91 individuals as previously described [6, 35]. Cases were evaluated from the Veterans Affairs-Boston University-Concussion Legacy Foundation (VA-BU-CLF), Framingham Heart Study (FHS), and Boston University Alzheimer’s Disease Center (BU ADC) brain banks. Cases were assessed for neurodegenerative disease using well-established criteria for AD [7, 20, 34], neocortical Lewy body disease (LBD) [31], frontal temporal lobar degeneration (FTLD) [27], motor neuron disease (MND) [26], and CTE [1, 6, 29]. Neuropathological evaluation occurred blinded to the clinical evaluation and was reviewed by four neuropathologists (AM, TS, VA, BH). Discrepancies in diagnosis were resolved by consensus conference. Two groups of cases were used in the current study: (1) individuals with a history of exposure to repetitive head trauma and a neuropathologic diagnosis of CTE or (2) individuals without exposure to repetitive head trauma, with neuropathologic changes consistent with AD, and a negative diagnosis of CTE. Cases were included into the CTE group based on the presence of available tissue and receiving a diagnosis for CTE while also a negative diagnosis for LBD, FTLD, MND, and AD. CTE cases were then divided based on McKee staging criteria: 10 cases were found to have CTE II, 15 had CTE III, and 16 had CTE IV [1]. No stage I CTE cases were used for the current study as no tau pathology is typically found in the hippocampus during that stage. Cases were included in the AD group based having no history of repetitive head trauma, a Braak stage ≥ 1, and a CERAD score ≥ 1. CERAD was scored using Bielschowsky silver stains. Additionally, cases in the AD group received a negative diagnosis of CTE, LBD, FTLD, and MND. Cases in the AD group were then grouped by Braak stage where AD Braak I-II had 8 cases, AD Braak III-IV had 21 cases, and AD Braak V-VI had 21 cases. A detailed breakdown of the total sample size, gender, age at death, and CERAD neuritic amyloid score can be found in Table 1. Next of kin provide written consent for participation and donation. Methods were approved and carried out in accordance with the institutional review boards from Boston University School of Medicine and the Edith Nourse Rogers Memorial Veterans Hospital, Bedford, MA.

**Table 1:** Demographic details

	n	Gender (m/f)	Age at Death (years)	CERAD
CTE II	10	10/0	45.5 ± 14.4	0.1 ± 0.3
CTE III	15	15/0	67.7 ± 12.7	0.2 ± 0.4
CTE IV	16	16/0	74.81 ± 7.7	0.6 ± 0.6
AD Braak I-II	8	5/3	87.4 ± 10.1	1.0 ± 0.0
AD Braak III-IV	21	12/9	86.1 ± 7.4	1.9 ± 0.8
AD Braak V-VI	21	11/10	83.1 ± 5.7	2.2 ± 0.9

Data expressed as Mean ± Standard Deviation

### Staining and analysis

Brain tissue was taken from the posterior hippocampus at the level of the lateral geniculate nucleus, processed, and stained as previously described [8]. Briefly, sections were incubated with antibodies to RD3 (3R Tau) (generous gift of Rohan de Silva, 1:6000, Clone 8E6/C11), ET3 (4R Tau) (generous gift of Peter Davis [12], 1:200), anti-PHF-Tau (AT8) (Pierce Endogen, 1:2000), and DAPI. The two tau isoform antibodies, RD3 and ET3, were selected based on the success of past studies examining the individual isoforms [8]. Sections were subjected to linear unmixing, background correction, and visualized with an Akoya Bioscience Vectra Polaris Digital Slide Scanner and inForm (Akoya Bioscience). Images were analyzed using HALO (Indica Laboratory). The hippocampus was subdivided into CA4, CA2/3, CA1, Subiculum, temporal cortex grey matter and temporal cortex white matter. CA2 and CA3 were combined into one field due to the lack of a definitive or clearly distinguishable boarder between the two. The temporal cortex grey matter was defined as the grey matter from the end of the subiculum to the first temporal sulcus. The temporal cortex white matter was defined as all the white matter that was present in the section that did not extend beyond the most dorsal aspect of the hippocampus. Counting of individual 3R and 4R containing cells was accomplished by training a HALO automated intelligence (AI) algorithm. Separate HALO AI nuclear segmentator and nuclear phenotyper algorithms were trained to identify all 3R and 4R positive cells. Each cell had to colocalize with AT8. Only cell bodies were analyzed. Each algorithm was trained on over 500 cells. The AI were then allowed to iterate for over 50,000 cycles before use on annotated images. AI was validated through visual inspection of results to confirm AI positive cell calls were correct. Neuropathologists (TS, AM) were consulted on AI training and validation. This method was chosen over stereology since pathology could be patchy or sparse and it was possible to miss tau containing cells if the whole medial temporal lobe was not assessed. Counts of tau containing cells were then standardized to total area measured for a final density value.

To determine the relative burden of each isoform in individual subfields compared to the whole hippocampus. The relative parentage of either 3R or 4R tau was calculated by taking the individual subfield counts for each isoform then dividing them by the sum total of the 3R or 4R counts for CA4, CA2/3, CA1, and the subiculum for each case.

Cases in the UNITE brain bank were also assessed for severity of extracellular tau (i.e. ghost tangle) pathology present in the hippocampus based on previous methods [30]. Bielschowsky silver stains were used to identify ghost tangle pathology. Neuropathologists noted a binary present/absent answer if more than 50% of the hippocampal neurons were ghost tangles.

### Statistics

Statistical analysis was performed using SPSS (version 24, IBM) and Prism (version 9, Graphpad Software). To determine if 4R or 3R tau was higher in each case, two-way ANOVAs with a Bonferroni post-test were run within each disease group. One sample t-tests were used to determine if the 4R/3R ratio was significantly different than an even ratio score of 1. Ordinal regression analysis was used to measure if the 4R or 3R tau densities compared to CERAD scores independently of age at death. Two-way ANOVAs were used to compare the density of each isoform across each hippocampal subfield within each disease. A One-way ANOVA was used to compare the relative percentages of tau in each subfield. To compare the different densities of 3R and 4R tau within a subregion, Pairwise Wilcoxon Rank Sum Tests were performed on each region. Values reported for pairwise comparisons are the FDR adjusted p-values. A receiver operator characteristic curve analysis was used to determine which subfield and which tau isoform had the highest specificity and sensitivity to distinguish AD and CTE. Finally, binary logistic regression was used among CTE cases to determine if age at death, 3R tau density, or 4R tau density correlated with a present/absent variable on if ghost tangle pathology was observed in 50% or greater hippocampal neurons.

## Results

### Tau was found in different densities and distributions within hippocampal subfields in each disease

**Fig. 1:**
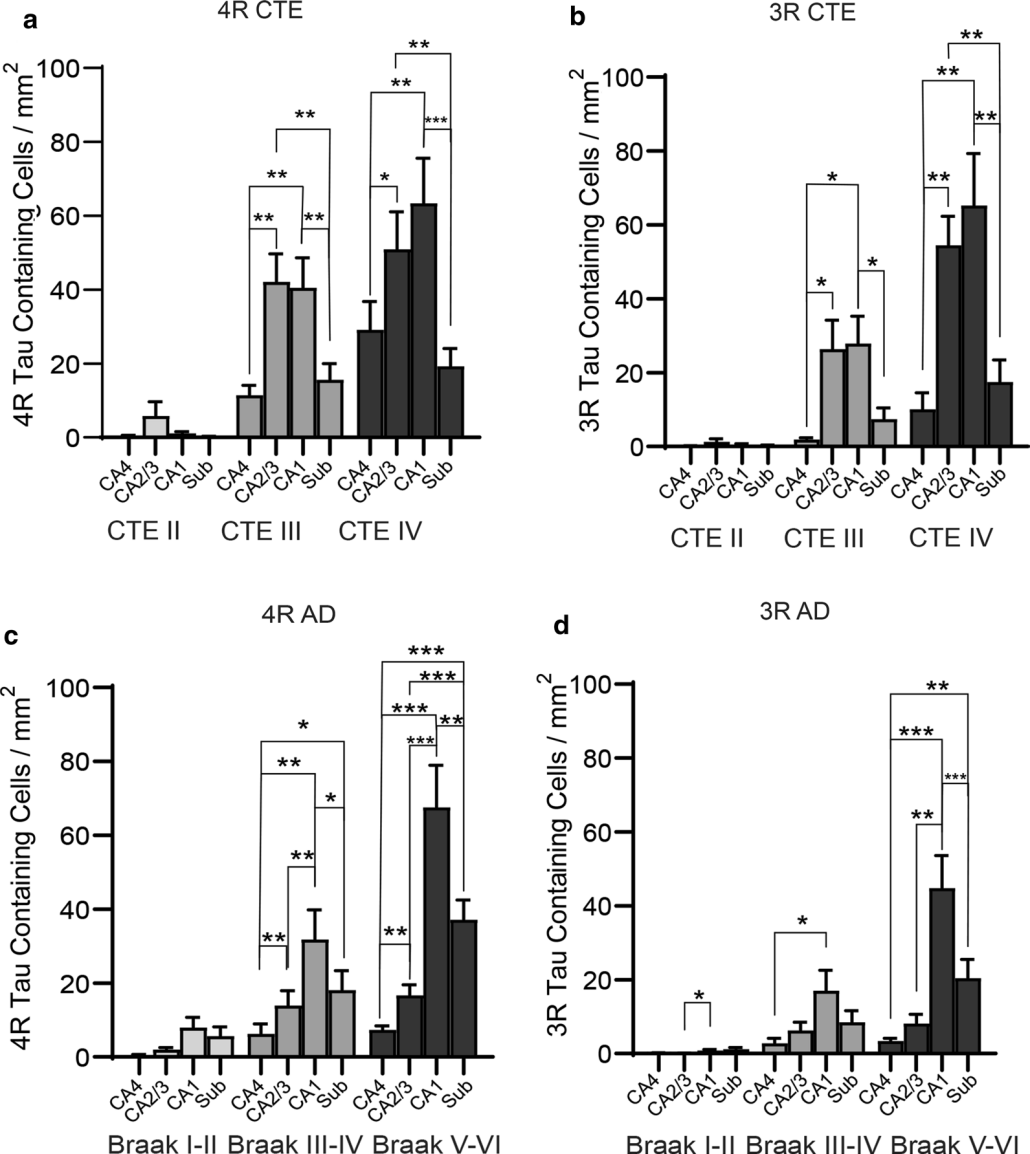
The relative density of tau isoforms found in each hippocampal subfield changes across each disease. Tau isoform densities were analyzed across each hippocampal subfield and compared within each disease to determine proportional differences in tau load. Quantitation of the **a** 4R and **b** 3R overall tau densities found in CTE. **c** 4R tau and **d** 3R tau was also analyzed in AD. Two-way ANOVA analysis was used for statistics. *p < 0.05, **p < 0.01, ***p < 0.001

To determine if specific hippocampal subregions had preferential accumulation of tau and if those increases are altered across disease, the hippocampus was subdivided into 4 fields (CA4, CA2/3, CA1, and the Subiculum), and tau isoform counts were performed (Fig. 1).

CTE: For CTE, no significant difference across the subfields was observed in CTE II. However, 2 out of 10 CTE II cases had a CA2/3 4R tau density of 35.58 and 20.25 cell/mm^2^ placing them at similar levels to CTE III cases. Using two-way ANOVA analysis CA4, CA1, and subiculum levels were comparable to other CTE II cases. In CTE III and IV, CA2/3 and CA1 had higher 4R tau densities than CA4 and subiculum (CTE III *p* = 0.002 and *p* = 0.0047, CTE IV *p* = 0.043 and *p* = 0.0008). CA1 and CA2/3 had similar 4R tau densities throughout CTE (CTE III *p* = 0.99, CTE IV *p* = 0.40) (Fig. 1a). Similar changes in CTE hippocampal subregions were seen with 3R densities as well (Fig. 1b).

AD: In AD using two-way ANOVA analysis, no significant differences were observed in AD Braak I-II for 4R tau (Fig. 1c). However, more 3R tau was observed in CA1 compared to CA2/3 (*p* = 0.32) (Fig. 1e). Unlike CTE, 4R tau density was increased in CA1 compared to all other regions in AD Braak III-IV and 5–6 (CA4 *p* = 0.0018, CA2/3 *p* = 0.0039, Sub *p* = 0.0418) (Fig. 1c). Also, unlike CTE, the subiculum had more 4R tau compared to CA4 in AD Braak III-IV (*p* = 0.016), and both CA4 (*p* < 0.0001) and CA2/3 (*p* = 0.0008) in AD Braak V-VI (Fig. 1d). Although less tau containing cells were observed, similar changes were also found in the 3R tau isoform densities across AD hippocampal subfields (Fig. 1d).

**Fig. 2:**
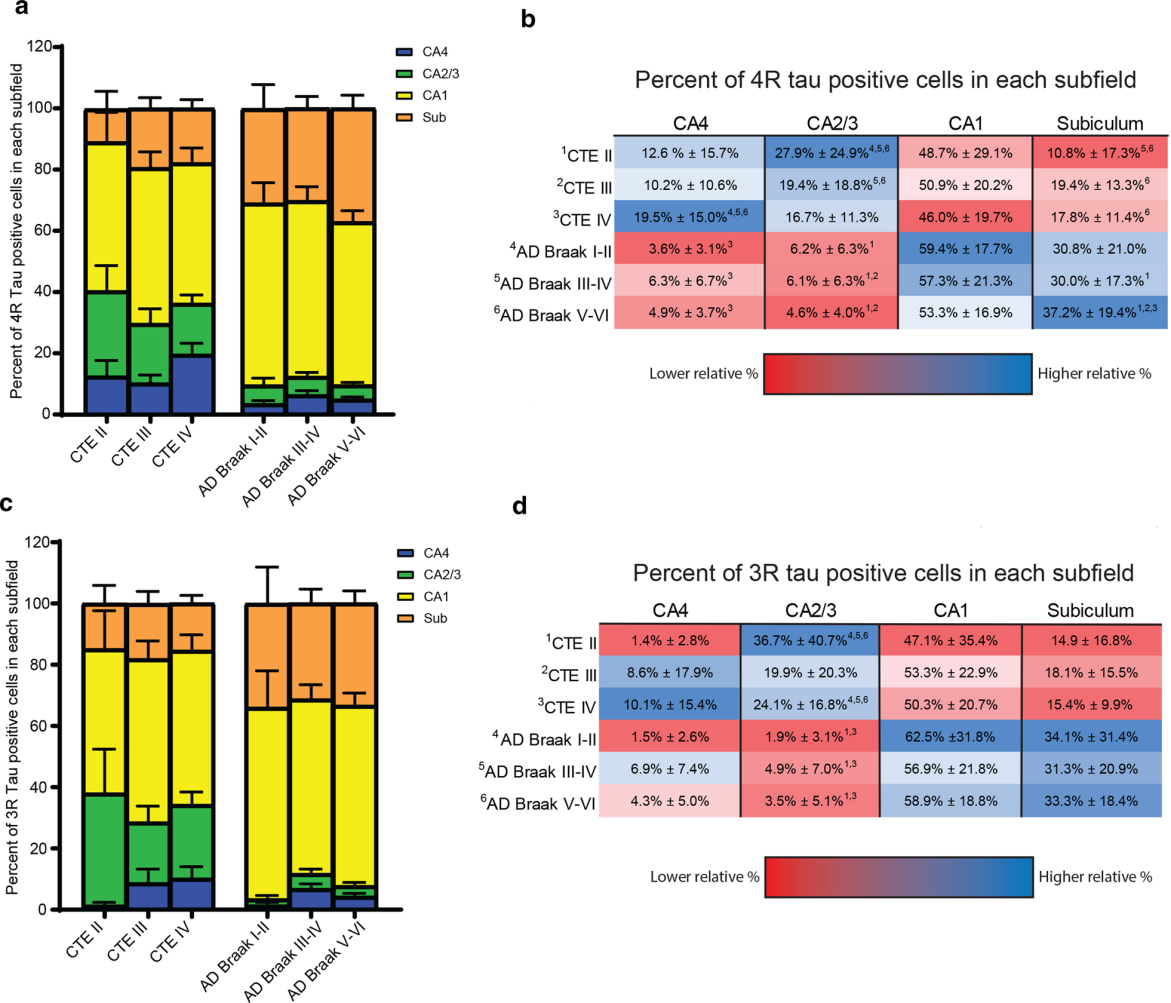
The relative percentage of tau in each hippocampal subfield is altered in AD and CTE. The relative amount of tau found in each hippocampal subfield was analyzed between AD and CTE. Percentages were calculated by dividing each region by the sum total of all the 3R or 4R tau found in CA4, CA2/3, CA1, and the subiculum. **a, c** Representative graph of the relative percentages of **a** 4R tau and 3R **c** isoforms found in AD and CTE. Percentages are divided based on hippocampal region. Error bars represent standard error of the mean. **b, d** The relative mean percentages ± standard deviation found in each subfield for **b** 4R and **d** 3R. The top 3 percentages in each subfield are highlighted in blue, while the bottom 3 percentages for each region are in red. Superscript text next to percentage denotes which disease group that value is significantly different from. See left side of pathology groups for corresponding superscript value. Statistics generated using a One-way ANOVA

To observe if the relative distribution of tau in each subfield was similar or different between AD and CTE, the relative percentage of tau containing cells in each subfield compared to the total number within all hippocampal subfields was calculated (Fig. 2). One-way ANOVA analysis demonstrated CTE had a higher relative percentage of 4R tau found in CA2/3 in stages II and III (*p* < 0.05), but not stage IV when compared to AD (Fig. 2a,b). CTE IV was also found to have a higher relative percentage of 4R tau compared to AD in CA4 (Fig. 2a,b) (*p* < 0.01). The subiculum had a greater percentage of 4R tau positive cells in AD compared to CTE (*p* < 0.05) (Fig. 2a,b). While all three AD groups had a higher relative percentage of 4R tau in CA1 compared to CTE, the data did not reach statistical significance (Fig. 2b). The relative distribution 3R tau was similar to 4R (Fig. 2c,d). The only difference being minimal (1.4% ± 2.8%) 3R tau was found in CA4 compared to the other subfields.

### 3R and 4R tau was expressed at different levels in the hippocampus in AD and CTE

**Fig. 3:**
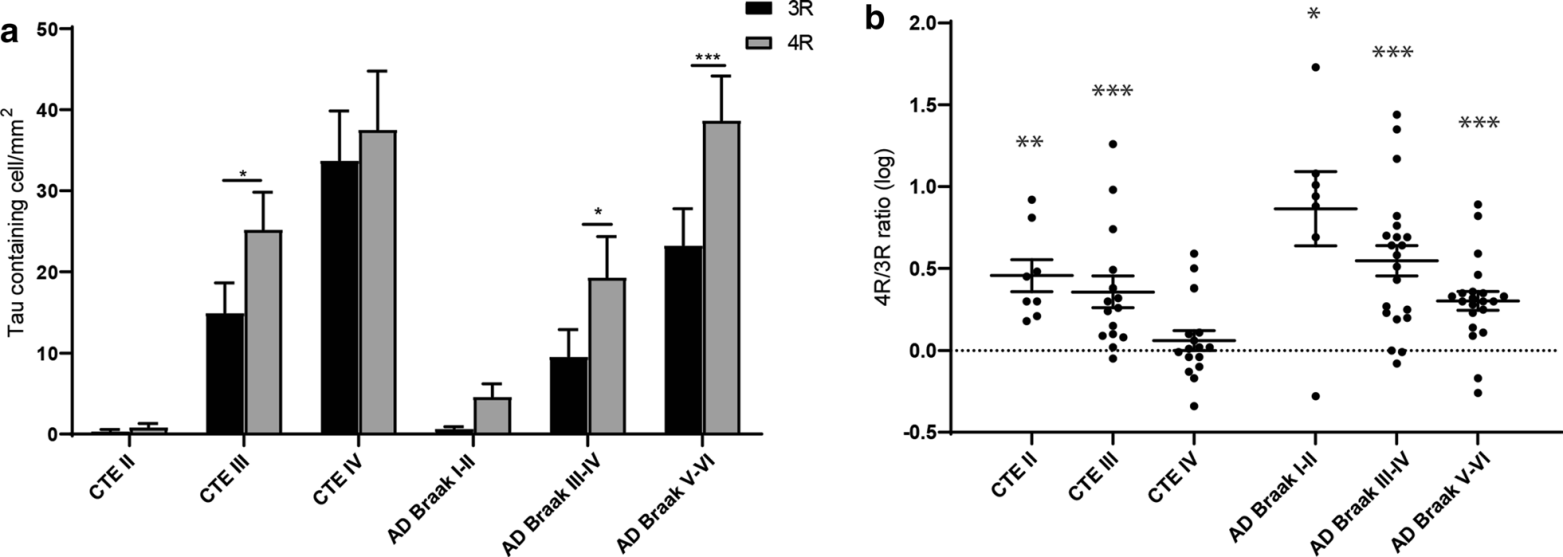
Total hippocampus analysis of 3R and 4R tau isoforms show shifts in 4R/3R phenotype over disease course. The total density of 3R and 4R tau containing cells across CA4, CA2/3, CA1, and the subiculum was analyzed across CTE and AD. **a** Comparative analysis of 3R and 4R tau isoforms within each disease. Paired two-way ANOVAs were used within each disease group to determine altered 3R/4R levels. **b** Analysis of the 4R/3R tau isoform ratio across each disease. Each dot represents a single case. A one sample T-test was used to determine in each ratio was significantly different than an even ratio value of 1. Dotted line represents a ratio value of 1. *p < 0.05, **p < 0.01, ***p < 0.001

The relative proportion of 3R or 4R in each disease was then analyzed to determine what isoform was predominant and if there was a shift during the course of disease (Fig. 3). Using a two-way ANOVA, a higher density of 4R compared to 3R tau was observed in CTE III (*p* = 0.036), AD Braak III-IV (*p* = 0.026), AD Braak V-VI (*p* = 0.0002). Notably, there was no difference in the 3R and 4R levels in CTE IV (*p* = 0.682) (Fig. 3a). When examining the ratio of 4R/3R across the disease groups using a one sample t-test, only CTE IV (*p* = 0.495) did not have a ratio significantly different than 1 (even 4R/3R ratio) (Fig. 3b). Using the individual ratio values, 7 out of 16 (43%) CTE IV cases had 4R/3R ratio values below 1, demonstrating more 3R tau was present than 4R. CTE IV had significantly more cases under a 4R/3R ratio than all other groups (*p* < 0.05). CTE III had 1 out of 15 (7%), AD Braak I-II had 1 out of 7 (14%), AD Braak III-IV has 2 out of 21 (10%), AD Braak V-VI had 2 out of 21 (10%). All cases had at least one 3R tau containing cells except 2 CTE II (20%) and 1 AD Braak I-II (13%).

### Comparison of the unique densities of each isoform in the hippocampal subfields across AD and CTE

**Fig. 4:**
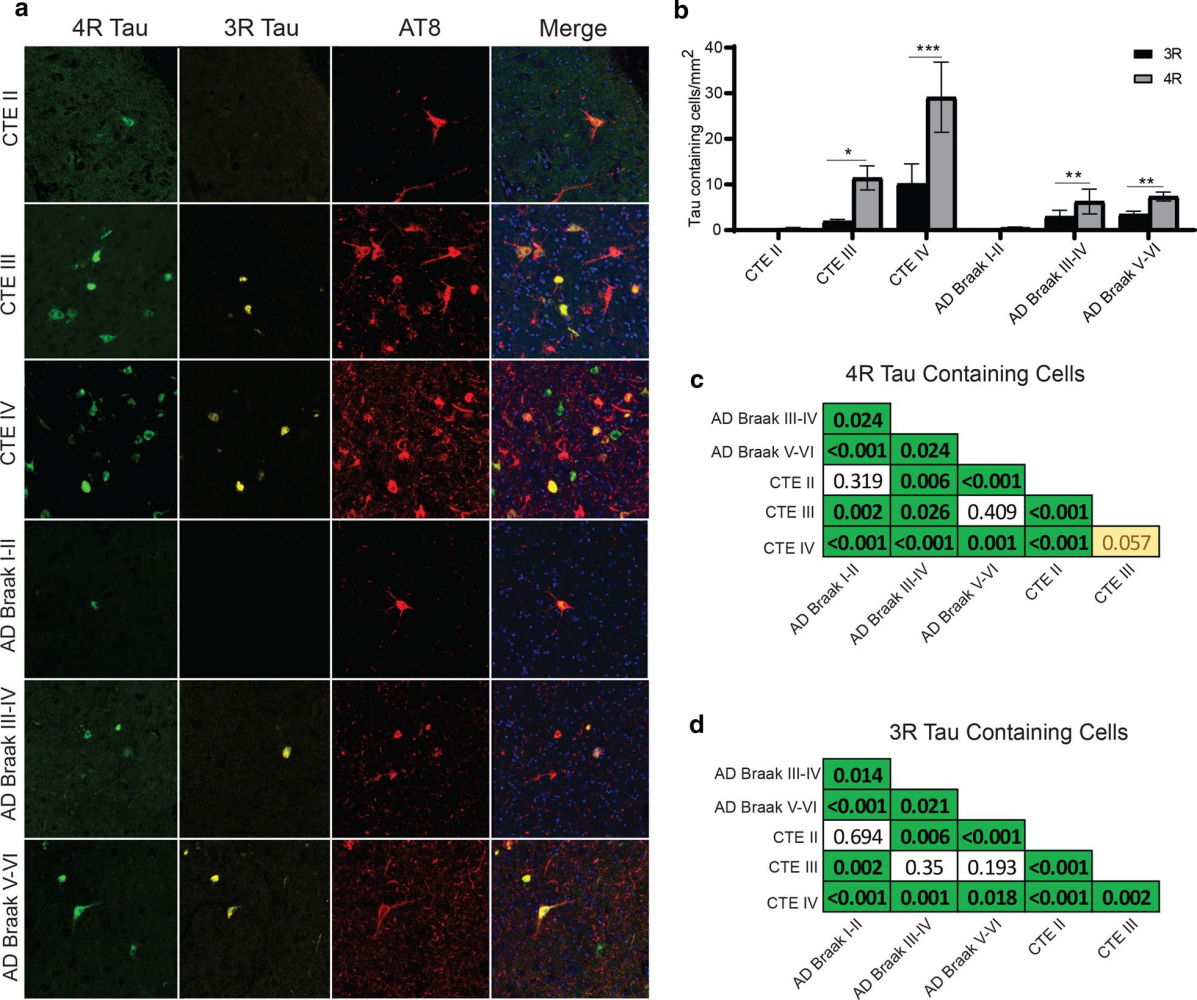
Comparison of 3R and 4R tau isoforms in CA4. **a** Representative image of 3R, 4R, and AT8 staining found in the hippocampal subfield CA4 across each disease. **b** Comparative analysis of the relative 3R and 4R tau isoforms density found in each disease. Paired two-way ANOVAs were used within each disease group to determine altered 3R/4R levels. *p < 0.05, **p < 0.01, ***p < 0.001. **c, d** Pairwise comparison of the **c** 4R and **d** 3R tau density between each pathologic group using a Pairwise Wilcoxon Rank Sum Tests. Values are FDR adjusted significance score. Green boxes represent significant values. Yellow Boxes are scores that are trending towards significance

**Fig. 5:**
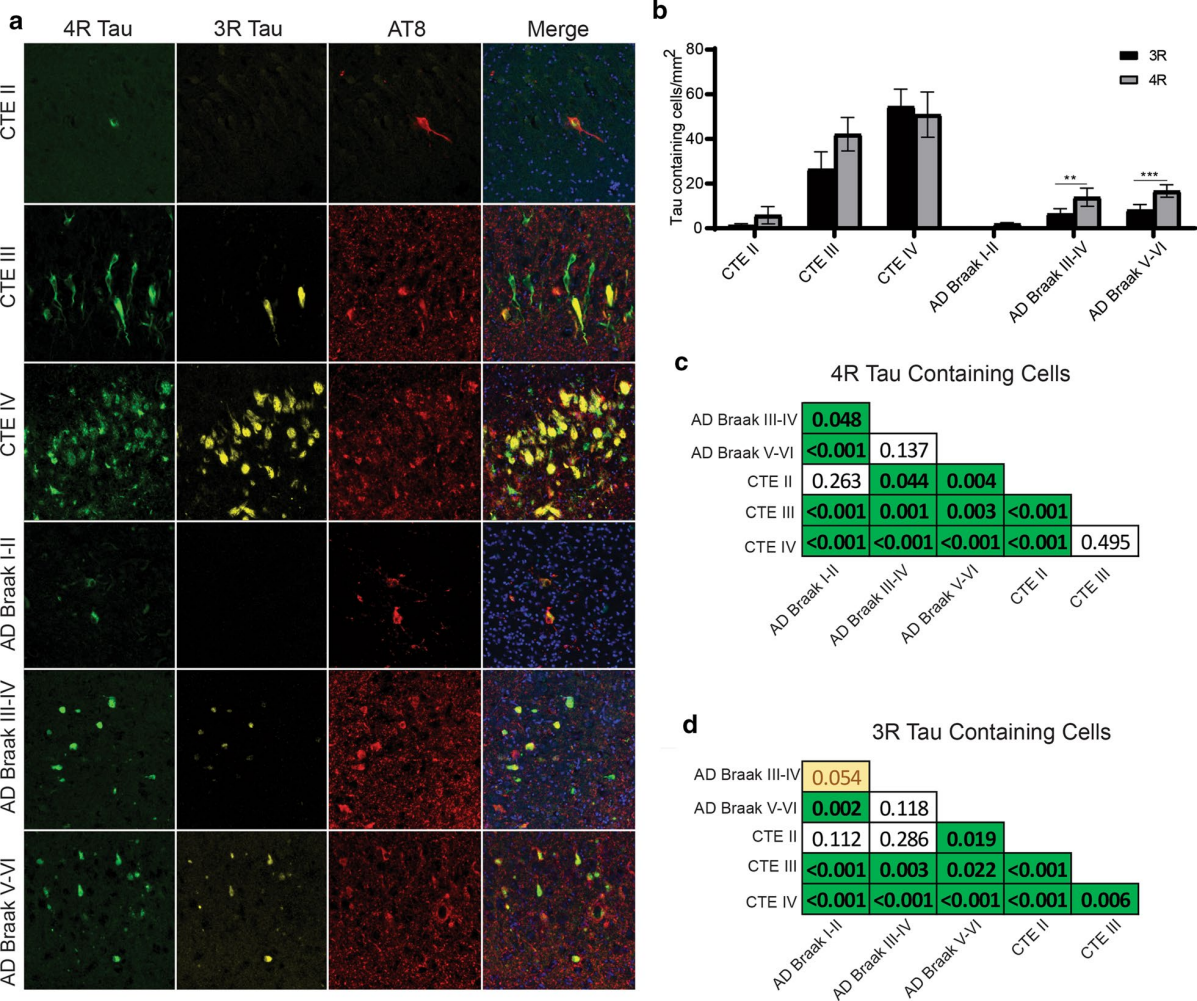
Comparison of 3R and 4R tau isoforms in CA2/3. **a** Representative image of 3R, 4R, and AT8 staining found in the hippocampal subfield CA2/3 across each disease. **b** Comparative analysis of the relative 3R and 4R tau isoforms density found in each disease. Paired two-way ANOVAs were used within each disease group to determine altered 3R/4R level. *p < 0.05, **p < 0.01, ***p < 0.001. **c, d** Pairwise comparison of the **c** 4R and **d** 3R tau density between each pathologic group using a Pairwise Wilcoxon Rank Sum Tests. Values are FDR adjusted significance score. Green boxes represent significant values. Yellow Boxes are scores that are trending towards significance

**Fig. 6:**
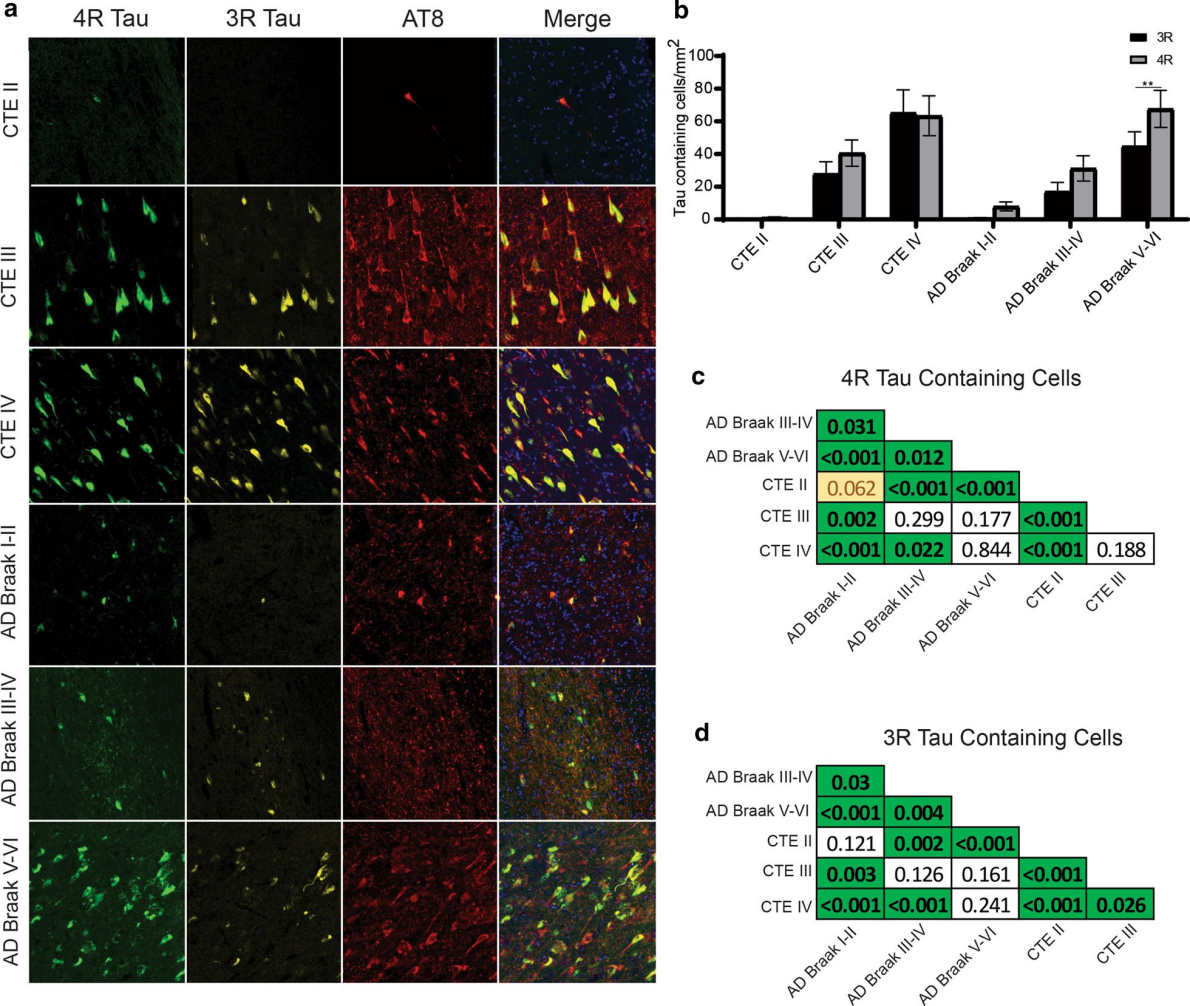
Comparison of 3R and 4R tau isoforms in CA1. **a** Representative image of 3R, 4R, and AT8 staining found in the hippocampal subfield CA1 across each disease. **b** Comparative analysis of the relative 3R and 4R tau isoforms density found in each disease. Paired two-way ANOVAs were used within each disease group to determine altered 3R/4R levels. *p < 0.05, **p < 0.01, ***p < 0.001. **c, d** Pairwise comparison of the **c** 4R and **d** 3R tau density between each pathologic group using a Pairwise Wilcoxon Rank Sum Tests. Values are FDR adjusted significance score. Green boxes represent significant values. Yellow Boxes are scores that are trending towards significance

**Fig. 7:**
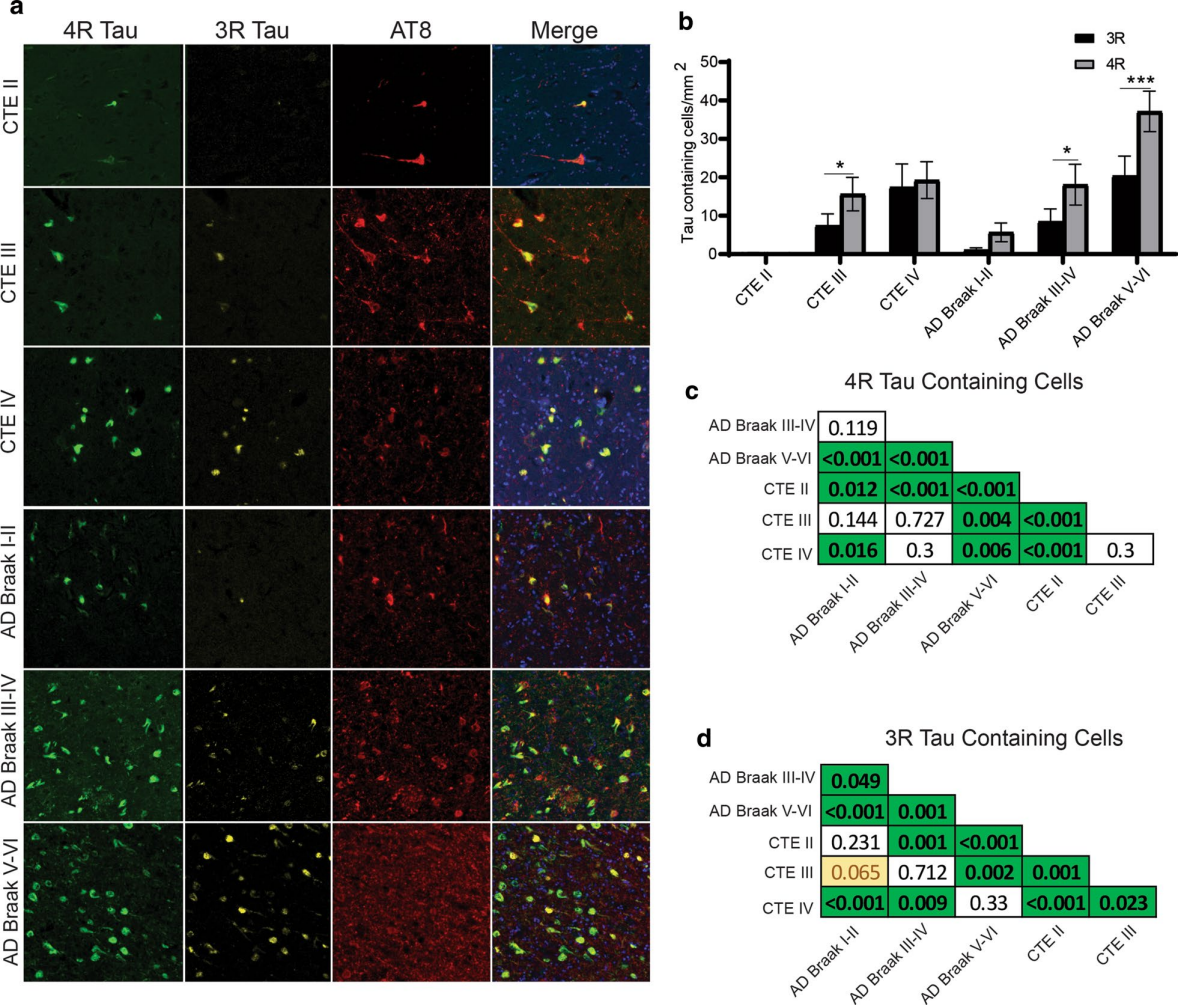
Comparison of 3R and 4R tau isoforms in the Subiculum **a** Representative image of 3R, 4R, and AT8 staining found in the subiculum across each disease. **b** Comparative analysis of the relative 3R and 4R tau isoforms density found in each disease. Paired two-way ANOVAs were used within each disease group to determine altered 3R/4R levels. *p < 0.05, **p < 0.01, ***p < 0.001. **c, d** Pairwise comparison of the **c** 4R and **d** 3R tau density between each pathologic group using a Pairwise Wilcoxon Rank Sum Tests. Values are FDR adjusted significance score. Green boxes represent significant values. Yellow Boxes are scores that are trending towards significance

**Fig. 8:**
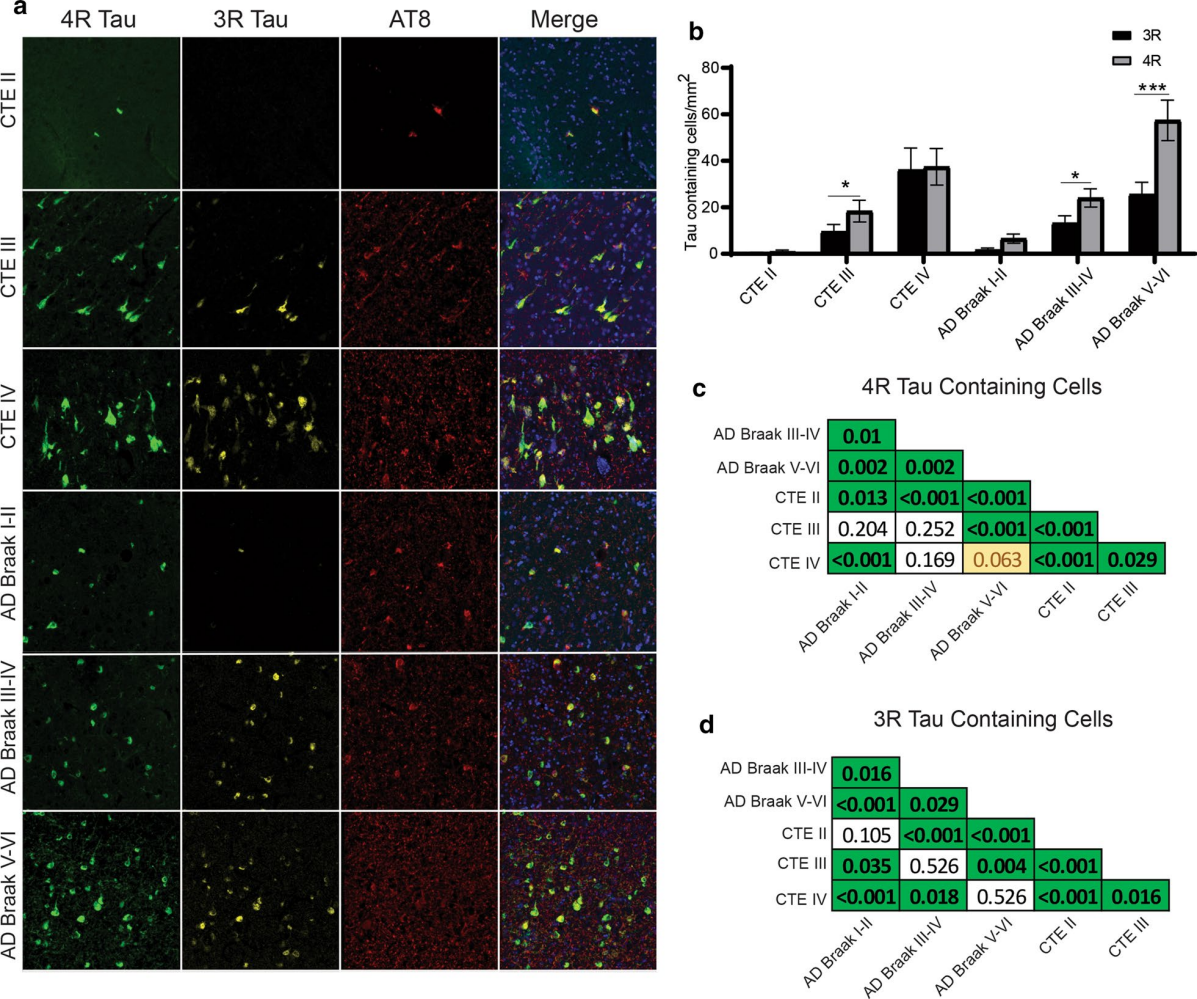
Comparison of 3R and 4R tau isoforms in Temporal Cortex Grey Matter. **a** Representative image of 3R, 4R, and AT8 staining found in the Temporal cortex grey across each disease. **b** Comparative analysis of the relative 3R and 4R tau isoforms density found in each disease. Paired two-way ANOVAs were used within each disease group to determine altered 3R/4R levels. *p < 0.05, **p < 0.01, ***p < 0.001. **c, d** Pairwise comparison of the **c** 4R and **d** 3R tau density between each pathologic group using a Pairwise Wilcoxon Rank Sum Tests. Values are FDR adjusted significance score. Green boxes represent significant values. Yellow Boxes are scores that are trending towards significance

**Fig. 9:**
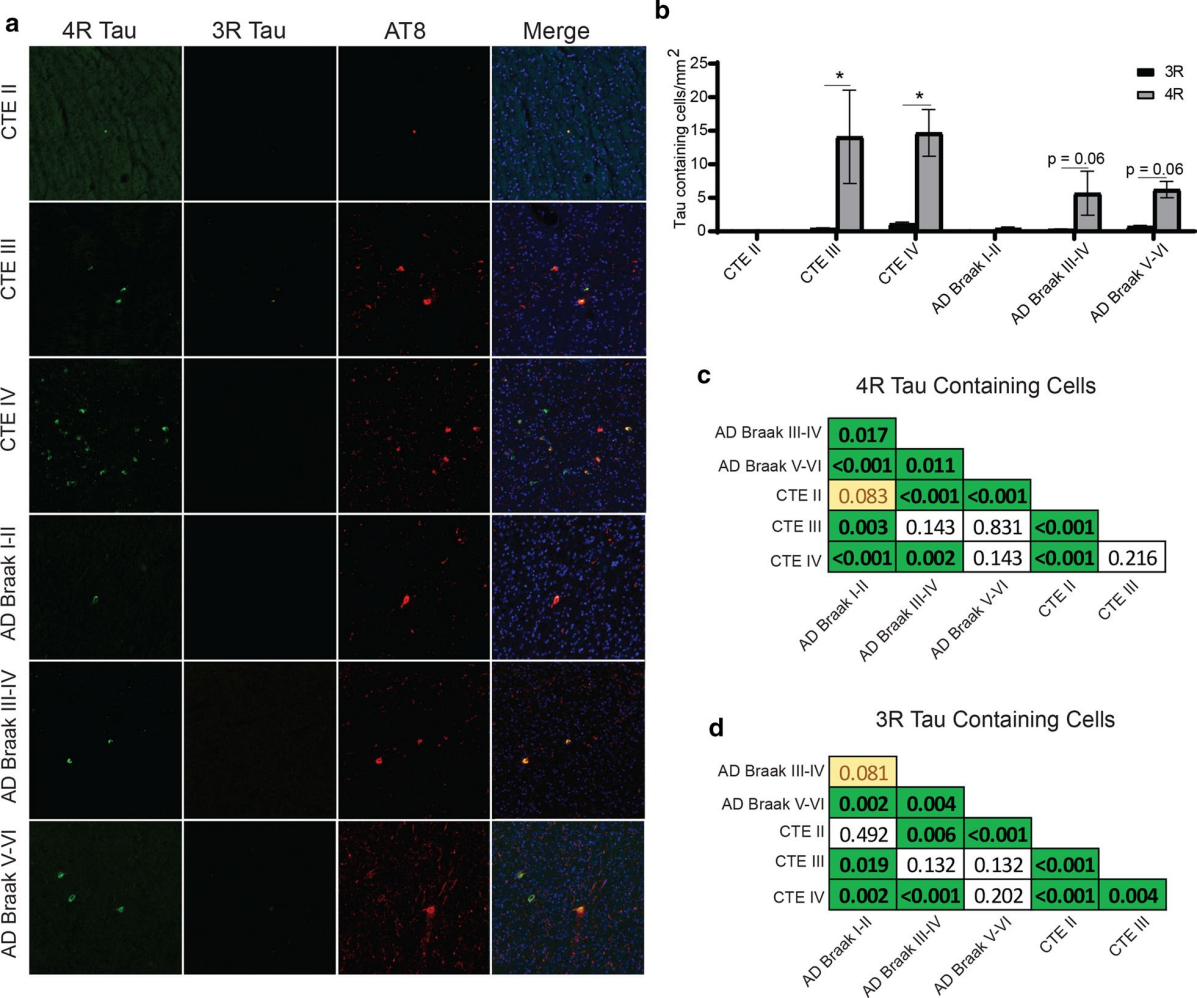
Comparison of 3R and 4R tau isoforms in the Temporal Cortex White Matter. **a** Representative image of 3R, 4R, and AT8 staining found in the temporal cortex white matter across each disease. **b** Comparative analysis of the relative 3R and 4R tau isoforms density found in each disease. Paired two-way ANOVAs were used within each disease group to determine altered 3R/4R levels. *p < 0.05, **p < 0.01, ***p < 0.001. **c, d** Pairwise comparison of the **c** 4R and **d** 3R tau density between each pathologic group using a Pairwise Wilcoxon Rank Sum Tests. Values are FDR adjusted significance score. Green boxes represent significant values. Yellow Boxes are scores that are trending towards significance

The overall densities of each isoform were then directly compared between AD and CTE across the four hippocampal subfields, in additional to the temporal cortex grey matter and temporal cortex white matter (Figs. 4, 5, 6, 7, 8, and 9).

### CA4 Subfield

In CA4, paired two-way ANOVAs demonstrated that 4R tau was elevated compared to 3R tau in CTE III (*p* = 0.0422), CTE IV (*p* < 0.0001), AD Braak III-IV (*p* = 0.0088), and AD Braak V-VI (*p* = 0.0014) (Fig. 4a,b). Pairwise Wilcoxon rank sum tests were then used to compare the CA4 4R (Fig. 4c) and 3R (Fig. 4d) densities between each pathologic group. The full list of CA4 pairwise comparisons can be found in Fig. 4c and d. Overall, cases with stage III or IV CTE, were found to have higher levels of 4R tau when compared to almost all other cases. Only AD Braak V-VI had comparable levels of 4R to CTE III, while CTE IV 4R tau was higher than all other groups. For 3R densities, CTE IV contained the highest overall compared to all the groups.

### CA2/3 Subfield

Unlike CA4, paired two-way ANOVAs demonstrated that similar levels of 3R and 4R were observed in CA2/3 in CTE (Fig. 5a,b). However, AD Braak III-IV (*p* = 0.0041) and AD Braak V-VI (*p* = 0.0009) had more 4R than 3R (Fig. 5b). When examining across each group (full list of pairwise comparisons are present in Fig. 5c,d), both CTE III and CTE IV had significantly higher levels of 4R compared to all other groups. When examining 3R densities, CTE III and IV also had elevated levels compared to AD.

### CA1 Subfield

CA1 was the hippocampal subfield next analyzed (Fig. 6). Using paired two-way ANOVAs, only AD Braak V-VI had significantly more 4R tau than 3R tau (Fig. 6a,b). Pairwise comparison between all the groups demonstrated CTE and AD had similar levels of 4R (Fig. 6c). AD and CTE also had similar levels of 3R (Fig. 6d). The full list of CA1 pairwise comparisons can be found in Fig. 6c and d.

### Subiculum

When analyzing tau in the subiculum with paired two-way ANOVAs, it was observed that CTE III (*p* = 0.0256), AD Braak III-IV (*p* = 0.0271) and AD Braak V-VI (*p* < 0.0001) had elevated 4R tau levels compared to 3R tau (Fig. 7a,b). 4R was also increased in PART cases but did not meet significance. Pairwise comparisons between the groups revelated that AD Braak V-VI had the highest 4R densities. For 3R density, CTE IV has a higher density compared to AD Braak I-II and Braak III-IV, but not AD Braak V-VI (Fig. 7d). The full list of subiculum pairwise comparisons can be found in Fig. 7c and d.

### Temporal Cortex Grey Matter

In the Temporal cortex grey matter, using a paired two-way ANOVA, 4R tau was observed to be elevated in CTE III (*p* = 0.0344), AD Braak III-IV (*p* = 0.0175), and AD Braak V-VI (*p* < 0.0001) (Fig. 8a,b). Pairwise comparisons demonstrated AD Braak V-VI had the highest level of 4R compared to all other groups (Fig. 8c). 3R tau was elevated in CTE IV compared to AD Braak I-II and AD Braak III-IV, but not AD Braak V-VI (Fig. 8d).

### Temporal Cortex White Matter

Last, the temporal cortex white matter was examined (Fig. 9). Overall, paired two-way ANOVAs demonstrated that 4R tau was observed to be the primary isoform found in almost all the white matter in all cases (CTE III *p* = 0.0201, CTE IV *p* = 0.0169, AD Braak III-IV *p* = 0.06, and AD Braak V-VI *p* = 0.063) (Figure a,b). Although there was significant variation between each sample, CTE was observed to have more 4R and 3R tau compared to AD (Fig. 9c,d).

#### CA4 and CA2/3 were the most sensitive regions for a differential diagnosis

**Fig. 10:**
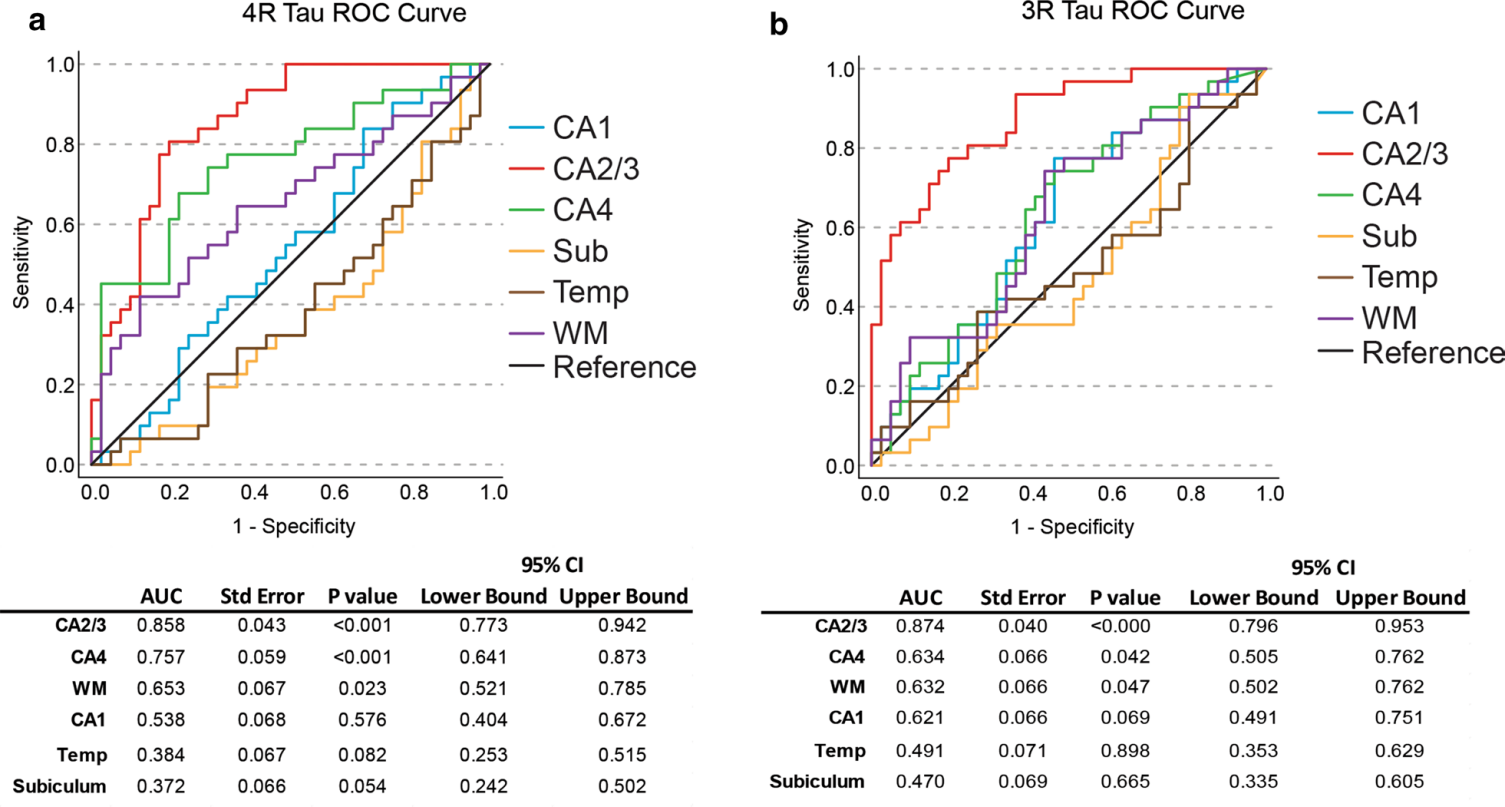
CA2/3 and CA4 demonstrate the highest ability to discriminate between AD and CTE. To determine which medial temporal lobe structure had the strongest ability to differentiate between severe AD (Braak III-VI) and high CTE (Stage III-IV), a receiver operator characteristic (ROC) curve was used to measure the individual fields across **a** 4R tau and **b** 3R tau. The area under curve (AUC) and relevant statistics for their respective regions are listed below the ROC curve. AUC are sorted from highest to lowest. Regions analyzed were CA1, CA2/3, CA4, Subiculum, Temporal cortex grey matter (Temp), and temporal cortex white matter (WM)

Receiver operator characteristic (ROC) curve analysis was utilized to determine which medial temporal lobe region was the most sensitive and specific for a neuropathologic diagnosis of High CTE (CTE III and CTE IV) vs severe AD (AD Braak III-VI) (Fig. 10). For 4R, CA2/3 had the highest area under the curve (AUC) of 0.858 (*p* < 0.001, std error = 0.043), followed by CA4 with 0.757 (*p* < 0.001, std error = 0.059). The white matter had the next highest AUC with 0.653 (*p* = 0.023, std error 0.067). The CA1, subiculum, and temporal cortex grey matter did not meet significance (Fig. 10a). For 3R, CA2/3 had the highest AUC with 0.874 (*p* < 0.001, std error = 0.04), followed by CA4 with 0.634 (*p* = 0.042, std error = 0.066) and then white matter with 0.632 (*p* = 0.047, std error = 0.066). CA1, subiculum, and temporal cortex did not meet significance (Fig. 10b).

#### 3R Tau correlateed with the increased ghost tangles in CTE

Finally, the density of 3R tau and the relation to the formation of ghost tangles found in the hippocampus in CTE was analyzed. Using a binary logistic regression analysis, it was observed that 3R tau density significantly correlated with a diagnosis that greater than 50% of hippocampal neurons were ghost tangles (Odds Ratio = 1.121, *p* = 0.022), independently of age at death (Odds Ratio = 1.103, *p* = 0.079) or 4R tau densities (Odds Ratio = 0.955, *p* = 0.135).

#### Amyloid does not influence 3R or 4R isoform density

To analyze if the presence of comorbid amyloid beta pathology influenced tau isoform expression, ordinal regression analysis was carried out (Table 2). In all cases, the CERAD score did not correlate with hippocampal 3R or 4R isoform density.

**Table 2:** Aβ does not correlate with 3R or 4R isoform expression

	CTE			AD		
	β	Std. Error	Sig	β	Std. Error	Sig
4R Tau Density	0.029	0.025	0.241	0.011	0.017	0.429
3R Tau Density	0.033	0.026	0.216	0.014	0.023	0.554

Analysis performed with ordinal regression using CERAD score as the dependent variable. Results are adjusted for age of death. CTE n = 41, AD n = 50

## Discussion

**Fig. 11:**
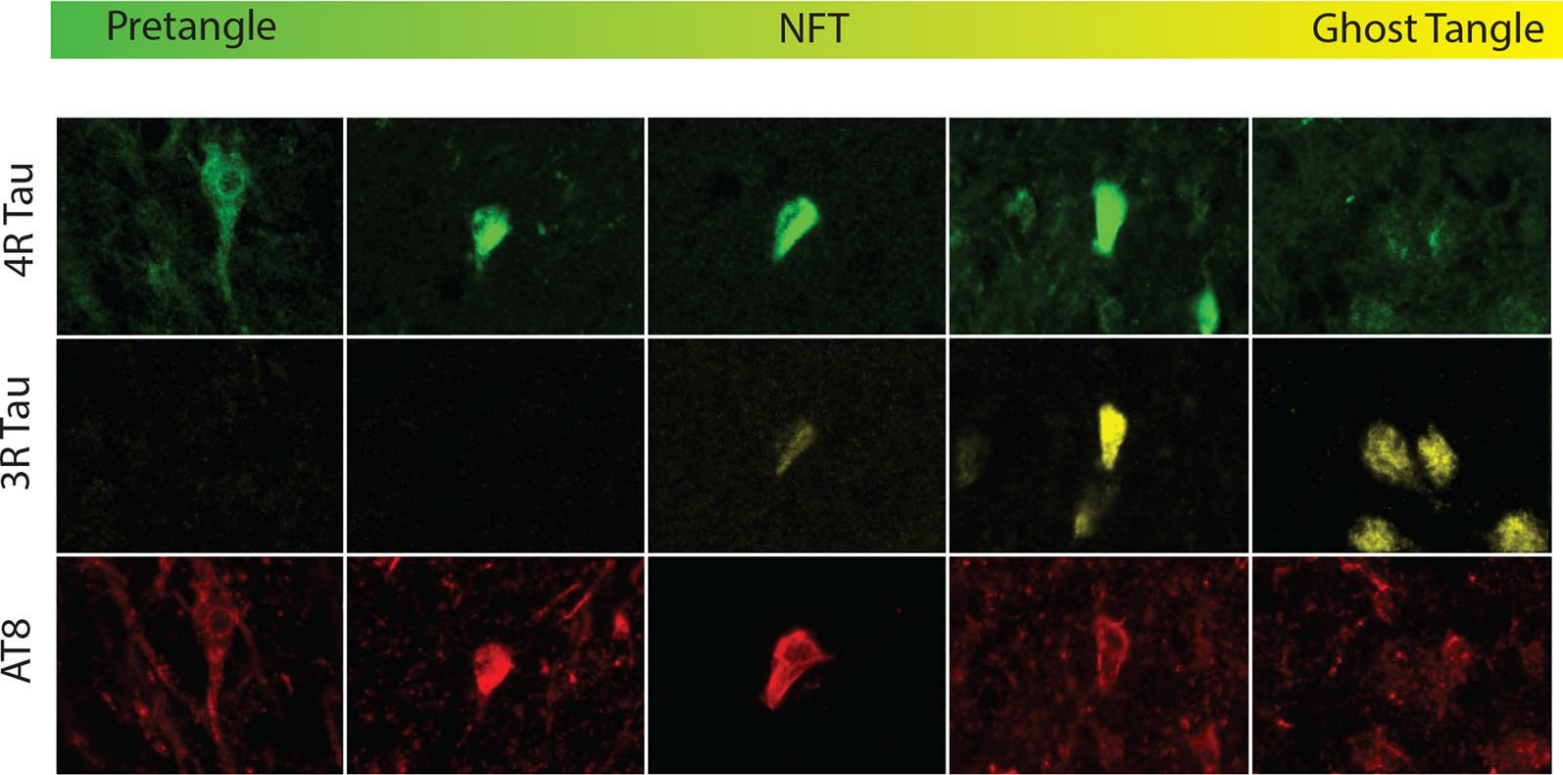
Working hypothesis model of 4R to 3R shift that occurs in mixed 3R/4R tauopathies. The current working hypothesis behind the evolution of the tau phenotype across disease severity is likely due to 3R strongly capturing ghost tangle pathology. Pretangles are believed to begin as a predominant 4R phenotype. Over the course of an NFT lifecycle, although the fibril structure is long lived, tau has been observed to be dynamic with monomers and oligomers constantly being added and replaced. Through a still unclear mechanism, there is a switch which results in more 3R tau being incorporated to the NFT as pathology becomes more severe. At the end stage, the ghost tangle is primarily composed of 3R tau. These findings have been seen across CTE and AD suggesting this phenotype is not specific to a unique pathology, rather, it is identifying something about the fundamental biology of mixed 3R/4R pathologies as a whole

Overall, it was observed that there was a high level of tau pathology diversity between AD and CTE when examining within the medial temporal lobe. Although both diseases are classified as a mixed 3R/4R tauopathy, 4R pathology appeared to be predominant in early disease, while 3R pathology surged in later stages. CTE demonstrated 43% of cases with severe pathology that had more 3R containing cells than 4R containing cells, while in AD, only 10% were seen in Braak stage V-VI. Significant differences in the densities of 3R and 4R tau isoforms were observed when individual hippocampal subfields were compared as well. In CTE, CA2/3 tau density was comparable to CA1 tau density. AD typically had higher CA1 and subiculum densities overall. Additionally, when looking at the relative spread of tau deposition between the four subfields, it was observed that CTE had a greater percentage of the total tau in CA4 and CA2/3 compared to AD, which was more CA1 and subiculum predominant. When examining white matter changes, 4R tau was the primary isoform but considerable variation and low overall densities prevented a full comparison. A ROC curve analysis demonstrated that higher relative levels of 4R tau found in CA2/3 and CA4, and 3R found in CA2/3 had highest specificity and sensitivity to differentially identify severe AD and CTE compared to the other subfields. Finally, the elevation in 3R tau isoform in late stage diseases was correlated to increased ghost tangle density and was likely related to the evolution of tau over the course of disease. These differences in isoform expression were found to be independent of Aβ pathology. Based on the results of the current study, a working hypothesis on the process of tau evolution seen in mixed 3R/4R pathologies is presented in Fig. 11.

The hippocampus is an important region for neuropathologic diagnoses and additional clarity of how disease affects specific subfields can aid future analyses. A major feature that best differentiated between AD and CTE was tau pathology found in CA4 and CA2/3 compared to tau pathology found in CA1 and the subiculum. CTE was observed to have a greater proportion of the total tau pathology, as well as a higher total amount in CA4 and CA2/3. While in AD, CA1 and the subiculum had a higher overall proportion of the total tau. Furthermore, ROC curve analysis suggested that using the 3R and 4R tau burden in CA2/3 and CA4 is a viable method to discriminate between the diseases. Interestingly, white matter 4R tau was also found to be different between the two as well. These observations have important ramifications for CTE as it strengthens previous qualitative neuropathologic observations and suggests additional diagnostic criteria for CTE when evaluating the hippocampus. Furthermore, the results highlight the possibility that CA4 and CA2/3 might have unknown vulnerabilities which makes them more susceptible to CTE pathology. Future work will be needed to determine if there are mechanisms or pathways present in those regions that are related to increase tau deposition which could be utilized for therapeutic targets.

The current results should be taken into context with the tau deposition pattern observed in other neuropathologies as well. CA2/3, followed by CA4, were observed to have the highest AUC and be the best discriminator between AD and CTE. However, a predominance of CA2 tau is observed in other diseases such as PART, CBD, PSP, and argyrophilic grain disease (AGD) [21, 33, 40]. It is unlikely that CBD, PSP, or AGD are driving the CA2/3 findings in the current study as any case with a comorbid disease diagnosis were excluded from the cohort. It is possible overlapping PART or other age-related pathology is present and driving some of the CTE related changes. However, PART is a disease only observed in the elderly [9] and the overall younger age of the CTE cohort suggests that the observed CA2/3 pathology occurs through an independent mechanism than PART. Altogether, while CA2/3 was observed to have the strongest ability to separate CTE and AD, CA4 might be a better overall identifier of CTE since the relative regional predominance of CA4 tau deposition appears to be unique to CTE. Future studies comparing the 3R and 4R deposition pattern and relative distribution across CTE and PART could further clarify the overlapping regional changes. Additionally, analysis of total neuronal densities for each subfield will be important to examine to better clarify the regional deposition of tau among unique diseases. It is likely that in regions such as CA4, that contain fewer total neurons than other subfields, a higher total percentage of neurons will contain tau in CTE compared CA1 or the subiculum well be observed, further defining CTE tau deposition from diseases like PART or AD.

Although the 3R and 4R tau isoforms have been traditionally used to classify neuropathologies, the current work suggests that the isoform dynamics might be not be as straight forward as once thought. It has become evident that 3R and 4R tau do not exist in a static state and can change over the course of disease. Recent work has suggested that although the tau inclusions are long lived, they are dynamic structures with consistent turnover of smaller parts [10]. Through still unclear mechanisms, the current results suggest that 3R tau becomes preferentially added into the tau fibril as disease severity increases. This could be attributed to the differential binding affinities between 3 and 4R tau [17]. Another hypothesis is that severe pathology triggers a change in the alternative MAPT splicing, resulting in more exon 10 exclusion, producing more 3R tau. However, there is a disconnect when speculating that 3R tau is related to more severe pathology, as diseases such as PSP or CBD do not aggregate 3R tau. This suggests that there is a fundamental difference in tau dynamics between 3R/4R mixed tau and 4R tau only diseases. It is likely this difference resides partly in the structure of the tau protein and fibril. Recent cryo-EM studies have identified that tau filaments have a unique structure among separate disease. CTE and AD were found to be most similar, while CBD was highly different [14, 43]. An important detail noted by the authors was that the filamental tau structure found in CBD (and likely other 4R tauopathies) has a much larger protein fold present. The authors hypothesize that this larger fold found in 4R only disease makes that variant of tau much harder to unfold or change and might be more resistant to disassembly [43]. Therefore, it is possible that once 4R only tau is present in a fibril, it is very resistant to disassembly and replacement by 3R. Interestingly, another difference was that in an in vitro system, 4R tau filaments did not recruit unbound 3R tau, while 3R/4R filaments did. This suggests that even if 3R was present in 4R tauopathies, it wouldn’t be recruited to the pathologic fibril. These results would partly explain the lack of an increase in 3R among more severe disease in 4R predominant tauopathies. It can also explain the lack of ghost tangles found in 4R only tauopathies, as the current study demonstrated a significant correlation between ghost tangles and 3R tau, but not 4R. It is unclear why 3R tau is more related to ghost tangle formation but one hypothesis could be related to that 3R tau and 4R tau have been demonstrated to recruit distinct protein binding partners [5, 24]. Each of these binding partners could trigger a unique downstream cascade that could result in divergent cellular apoptosis and death pathways. Future studies will be needed to determine if there are unique binding partners for specific isoforms of tau and how those binding partners might differentially contribute to neuropathology.

The evolution of 3R/4R tau in the hippocampus of CTE and AD has been previously shown in the frontal cortex in CTE [8] and in the hippocampus in AD [18]. This suggests that these findings are not specific to one disease or brain region but could be an inherent feature of all mixed tauopathies. These findings have important implications for biomarker and imaging studies utilizing single targets for specific tau isoforms. Individual PET tracers have had difficulty recognizing different isoforms of tau. A 4R tracer has challenges recognizing 3R/4R tau and vice versa [23]. The findings from the current study suggest that tau changes of the course of disease, and a “one tracer fits all” approach will miss parts of the disease. A mixed 3R/4R tracer is likely to miss early pathologic changes where the pathology is 4R predominant. Therefore, it is likely future PET studies might need to include multiple tracers to capture the full spectrum of disease. Additionally, the diversity of the tau isoform response could be novel biomarker targets to track disease progression. Cerebral spinal fluid tau levels are already commonly used to help identify possible neurodegenerative pathology in life [2]. Further refining these assays to capture the relative amounts of 3R or 4R tau could give additional clues to disease stage as well as type of disease. Ultimately, future biomarker and imaging studies will need to work in concert to provide a complete picture of the neuropathologic changes during life.

There are several limitations to the current study. The immunofluorescence staining relies on antibody specificity for select epitopes. Therefore, it is possible specific protein conformations or post-translational modifications, like acetylation, methylation, or ubiquitination might influence antibody binding. However, the observed increases in 3R and 4R tau were consistent with past biochemical and histologic studies, some of which use different sets of antibodies [8, 11, 12, 18]. This suggests the observed results were minimally affected by epitope modification. Post-translational modifications are an important area of research for tau deposition and future studies will be needed to determine possible modification related changes. Additionally, the current study did not examine individual cell types that contained tau. Both neurons and astrocytes can contain tau and the isoform dynamics might be altered in each. Previous work suggested that neurons contain 3R and 4R tau, while astrocytes only contained 4R. However, the astrocytic tau was more related to age [8]. As these findings were restricted to CTE, it is unclear if they translate to other neuropathologies. However, unlike the frontal cortex, astrocytic tau pathology found in the medial temporal lobe grey matter has been observed to be a relatively rare event [22]. Therefore, it is likely that the results of the current study are reflective of primarily neuronal changes. Additional cell type analysis might also help better refine the white matter changes as white matter astrocytic tau is sometimes a prominent feature of neurodegeneration. Although individual cell type analysis was beyond the scope of the current study, future work will be needed to determine if the isoform specific changes are due to unique populations of cellular populations. Additionally, cell loss and hippocampal atrophy are a feature of several neurodegenerative disease and could influence analyses. While the current study normalized all tau isoform counts to the area measured to account for changes in tissue size, future studies are needed to explore if cell loss and atrophy play a role in isoform expression. Other limitations include that the cohort that was used for the study represent a convenience sample limited to a pool of cases who voluntary donated and might not be representative of the population as a whole. A final limitation is the current study rely on the use of specific antibodies and certain epitopes of tau might be missed.

In conclusion, significant differences among 3R and 4R tau were observed in AD and CTE when examining the medial temporal lobe and hippocampal subfields. Tau deposition in the CA4 and CA2/3 hippocampal subfields were good discriminators of disease and will be important to add to future neuropathologic criteria. Furthermore, tau pathology was also observed to change over the course of disease. Early stage disease had higher levels of 4R while end stage disease had equal, if not more, 3R pathology. Higher densities of 3R were related to ghost tangles suggesting a mechanistic consequence of higher 3R tau isoforms. Overall, the work present here provides novel targets that may lead to biomarkers that can help track the progression of various neurodegenerative disease and better identifies future therapeutic strategies.

## Data Availability

The datasets generated for the current study are not publicly available due to ethical considerations of postmortem donation information, but are available from corresponding author through written request.
